# Discovery of sea urchin NGFFFamide receptor unites a bilaterian neuropeptide family

**DOI:** 10.1098/rsob.150030

**Published:** 2015-04-22

**Authors:** Dean C. Semmens, Isabel Beets, Matthew L. Rowe, Liisa M. Blowes, Paola Oliveri, Maurice R. Elphick

**Affiliations:** 1 School of Biological and Chemical Sciences, Queen Mary University of London, Mile End Road, London E1 4NS, UK; 2 Department of Biology, Functional Genomics and Proteomics Group, KU Leuven, Leuven, Belgium; 3 Department of Genetics, Evolution and Environment, University College London, Darwin Building, Gower Street, London WC1E 6BT, UK

**Keywords:** neuropeptide, evolution, receptor, neuropeptide-S, sea urchin, NGFFFamide

## Abstract

Neuropeptides are ancient regulators of physiology and behaviour, but reconstruction of neuropeptide evolution is often difficult owing to lack of sequence conservation. Here, we report that the receptor for the neuropeptide NGFFFamide in the sea urchin *Strongylocentrotus purpuratus* (phylum Echinodermata) is an orthologue of vertebrate neuropeptide-S (NPS) receptors and crustacean cardioactive peptide (CCAP) receptors. Importantly, this has facilitated reconstruction of the evolution of two bilaterian neuropeptide signalling systems. Genes encoding the precursor of a vasopressin/oxytocin-type neuropeptide and its receptor duplicated in a common ancestor of the Bilateria. One copy of the precursor retained ancestral features, as seen in highly conserved vasopressin/oxytocin–neurophysin-type precursors. The other copy diverged, but this took different courses in protostomes and deuterostomes. In protostomes, the occurrence of a disulfide bridge in neuropeptide product(s) of the precursor was retained, as in CCAP, but with loss of the neurophysin domain. In deuterostomes, we see the opposite scenario—the neuropeptides lost the disulfide bridge, and neurophysin was retained (as in the NGFFFamide precursor) but was subsequently lost in vertebrate NPS precursors. Thus, the sea urchin NGFFFamide precursor and receptor are ‘missing links’ in the evolutionary history of neuropeptides that control ecdysis in arthropods (CCAP) and regulate anxiety in humans (NPS).

## 2. Introduction

Sequencing of the human genome revealed the presence of many genes encoding G-protein coupled neuropeptide receptors (GPCRs) with unknown ligands—so-called orphan receptors [1]—and one of the first to be ‘deorphanized’ was GPR154. Comparison of the sequence of GPR154 with other GPCRs in humans revealed that it is most closely related to vasopressin/oxytocin (VP/OT)-type receptors, and therefore it was also referred to as vasopressin receptor-related receptor 1 (VRR1). However, when the pharmacological properties of VRR1/GPR154 were analysed it was found that VP/OT-type peptides are not ligands for this receptor [2]. Therefore, efforts were made to purify the endogenous ligand for this receptor from brain extracts, and it was found to be a 20-residue peptide with the sequence SFRNGVGTGMKKTSFQRAKS, which was named neuropeptide-S (NPS) on account of the N-terminal serine residue [3]. Interestingly, however, NPS does not share structural similarity with VP/OT-type peptides. Analysis of the physiological roles of NPS in rodents has revealed that it has an anxiolytic-like action, while also causing arousal [3]. Subsequently, it has been found that polymorphisms in the NPS receptor (NPSR) are associated with panic disorders in humans [4,5]. Thus, these findings indicate that NPS is an endogenous regulator of anxiety in humans and other mammals.

Investigation of the phylogenetic distribution of NPS and NPSR has revealed that genes encoding these proteins are present in tetrapod vertebrates but not in teleost fish or other non-tetrapod vertebrates, which suggested that the NPS–NPSR signalling system may have originated in a common ancestor of tetrapods [6]. However, a broader phylogenetic analysis reveals that orthologues of NPSR are not only present in tetrapod vertebrates, but are also found in invertebrates; for example, NPSR-related proteins are present in *Drosophila* and other insects [7,8]. Furthermore, the ligand that activates the NPSR-type protein in *Drosophila* has been identified as crustacean cardioactive peptide, or CCAP (PFCNAFTGCamide), a neuropeptide that controls ecdysis behaviour in insects [9–11]. Interestingly, NPS and CCAP share very little sequence similarity, and therefore the discovery that their receptors are orthologous was unexpected.

When CCAP was originally identified on account of its excitatory effect on crab hearts, it was noted that it shares superficial sequence similarity with VP/OT-type neuropeptides—in particular, the presence of a disulfide bridge between two cysteine residues [12]. CCAP precursor proteins do not, however, have a neurophysin domain, which is a characteristic feature of VP/OT-type precursors [13]. Nevertheless, the fact that CCAP receptors are paralogous to VP/OT-type receptors [8] suggests that CCAP and VP/OT-type peptides may have evolved from a common ancestral molecule. What is not clear, however, is the relationship between NPS and VP/OT-type peptides or CCAP. NPS shares no apparent structural similarity with VP/OT-type peptides, although it has been reported that NPS shares an FxN motif with CCAP [7,8].

A strategy to bridge understanding of relationships between neuropeptides in vertebrates (e.g. NPS) and neuropeptides in distantly related protostomian invertebrates such as insects (e.g. CCAP) is to analyse neuropeptide systems in deuterostomian invertebrates (e.g. cephalochordates, hemichordates, echinoderms), which are more closely related to vertebrates than protostomian invertebrates [14]. Analysis of genome sequence data has revealed that NPSR/CCAPR-related proteins are present in deuterostomian invertebrates [8,15]. Furthermore, we have discovered a gene in the cephalochordate *Branchiostoma floridae* that encodes a neuropeptide-type precursor protein containing two copies of an NPS-like peptide with the predicted amino acid sequence SFRNGVamide [16]. This peptide is identical to the bioactive N-terminal region of NPS [17], indicating that SFRNGVamide and NPS may have a common evolutionary origin. Homologues of the SFRNGVamide precursor are also present in non-chordate deuterostomian invertebrates—for example, the hemichordate *Saccoglossus kowalevskii* (acorn worm) and the echinoderm *Strongylocentrotus purpuratus* (sea urchin). The neuropeptides derived from these precursors have in common with SFRNGVamide (and NPS) the structural motif Asn–Gly (NG)—the *S. kowalevskii* precursor contains five copies of NGFWNamide and one copy of NGFYNamide, and the *S. purpuratus* precursor contains two copies of the putative neuropeptide NGFFFamide. Therefore, this family of neuropeptides in deuterostomian invertebrates has been designated as ‘NG peptides’ [16].

A surprising feature of NG peptide precursors in deuterostomian invertebrates is that they contain a neurophysin domain. Hitherto, neurophysins were thought to be a unique feature of VP/OT-type precursors, where they are required for axonal transport and secretion of neuropeptides [18]. Therefore, the discovery that NG peptides in deuterostomian invertebrates are derived from neurophysin-containing precursors changed the textbook perspective on neurophysins [16]. Furthermore, the presence of a neurophysin domain in NG peptide precursors provides a ‘missing link’ between NPS and VP/OT-type peptides. It suggests that an ancestral VP/OT-type precursor gene duplicated and one copy retained the highly conserved features of VP/OT-type precursors, whereas the other copy gave rise, on the one hand, to genes encoding NG peptide precursors in deuterostomian invertebrates, and on the other hand, to genes encoding CCAP-type neuropeptides in protostomian invertebrates. To address this hypothesis, the principal objective of the study presented here was to determine whether an NPSR-related receptor in a deuterostomian invertebrate species, the sea urchin *S. purpuratus*, is activated by NG peptides.

## 3. Material and methods

### 3.1. Mass spectrometry

Extracts of adult *S. purpuratus* were prepared by Dr Robert Burke at the University of Victoria (British Columbia, Canada). The test and viscera were homogenized separately in a Waring blender and then centrifuged (15 000*g* for 10 min). The supernatant was lyophilized overnight, and stored at −80°C prior to shipping to QMUL (UK). Peptides were extracted using an alcohol solvent (a 90 : 9 : 1 ratio of methanol : water : acetic acid), using an extraction protocol adapted from Husson *et al.* [19]. After removing the methanol solvent by evaporation, the extract was delipidated in a 1 : 1 ratio solution of ethyl acetate and *n-*hexane. Extracts were filtered using a C18 Sep-Pak cartridge (Waters), with solutions applied to the C18 column prepared in 0.1% formic acid. The column was washed with 40% acetonitrile, before loading 5 ml of extract, slowly washing with water, then 30% acetonitrile to remove pigment, before elution of the peptides with 80% acetonitrile, which was then removed by evaporation.

The HPLC–MS system used for peptide detection incorporated an Agilent 1100 Series pumping system, a Vydac C8 chromatography column (cat. no. 208TP54), an atmospheric pressure interface electrospray source, an ion trap mass analyser and a dynode-based system for ion detection. The nebulizer pressure was set to 40 psi, the flow rate was set to 0.5 ml min^−1^ and the injection volume set to 20 µl. Synthetic NGFFFamide (synthesized by the Advanced Biotechnology Centre, Imperial College London) was used as a standard for the optimization of mass spectrometry parameters. Synthetic NGFFFamide was found to elute at a concentration of 39–47% acetonitrile, at three different increasing flow rate gradients from 0.5% to 2% acetonitrile per minute. Thus, the detection of NGFFFamide in extracts of sea urchin tissue was guided by the elution point of the synthetic peptide run on an identical gradient.

### 3.2. Cloning of a cDNA encoding the *Strongylocentrotus purpuratus* neuropeptide-S/crustacean cardioactive peptide-type receptor

Using total cDNA from 72 h embryonic *S. purpuratus* as a template, the full-length cDNA of the *S. purpuratus* NPS/CCAP-type receptor, including 5′ and 3′ untranslated regions (UTR), was amplified by PCR using Phusion high-fidelity PCR master mix (NEB) and the oligos 5′-CCGACATAGAAAGTCATAG-3′ and 5′-GTCAGATCAGATATAGCAGT-3′. The PCR product was gel-extracted and purified using a QIAquick gel extraction kit (QIAgen) before being blunt-end cloned into a pBluescript SKII (+) vector (Agilent Technologies) cut with a *Eco*RV-HF restriction endonuclease (NEB). Subsequently, the full-length cDNA of the *S. purpuratus* NPS/CCAP-type receptor was subcloned into the eukaryotic expression vector pcDNA 3.1+ (Invitrogen) cut with *Bam*HI and *Apa*I restriction endonucleases (NEB) using the oligos 5′-cgggatccCACCATGGCGACACAAGTGA-3′ and 5′-atcgggcccCTACATCGGACTAGTAGTC-3′. The partial Kozak sequence (CACC) was incorporated immediately preceding the authentic initiation codon to optimize initiation of translation. The clone was then sequenced (Eurofins Genomics) from the T7 and pCR3.1-BGH-rev sequencing primer sites.

### 3.3. Pharmacological characterization of heterologously expressed *Strongylocentrotus purpuratus* neuropeptide-S/crustacean cardioactive peptide-type receptor

NGFFFamide and other synthetic NG peptides (NGFFYamide and NGIWYamide) were assessed as ligands for the *S. purpuratus* NPS/CCAP-type receptor using an *in vitro* calcium (Ca^2+^) mobilization assay that has been employed previously for deorphanization of invertebrate neuropeptide receptors [20,21]. Chinese hamster ovary (CHO)-K1 cells stably overexpressing the mitochondrial targeted apo-aequorin and the human Gα_16_ protein were used as a heterologous expression system. The cells were cultivated in Dulbecco's modified Eagle's medium/F-12 Ham (Sigma-Aldrich) supplemented with 10% fetal bovine serum (Sigma-Aldrich), 100 UI ml^−1^ of penicillin/streptomycin (Invitrogen), 250 µg ml^−1^ zeocin (Invitrogen) and 2.5 μg ml^−1^ amphotericin B (Sigma-Aldrich), in a humidified atmosphere of 5% CO_2_ in air at 37°C. Cells were transiently transfected with the *S. purpuratus* NPS/CCAP-type receptor/pcDNA 3.1+ construct using lipofectamine Ltx with plus reagent (Invitrogen) according to the manufacturer's instructions, whereas cells for negative control experiments were transfected with an empty pcDNA 3.1+ eukaryotic expression vector (Invitrogen). Two days post-transfection, cells were challenged with NG peptides (within the concentration range 10^−17^ to 2.5 × 10^−4^ M), and receptor activation at each peptide concentration was monitored by measuring Ca^2+^ levels for 30 s using a Mithras LB 940 luminometer (Berthold Technologies). Ca^2+^ responses were normalized to the total Ca^2+^ response monitored after addition of Triton X-100 (0.1%), and measured in triplicate in at least two independent experiments. Dose–response data were determined as relative (%) to the highest value (100% activation). Half-maximal effective concentration (EC_50_) values were calculated from dose–response curves, constructed with a computerized nonlinear regression analysis using a sigmoidal dose–response equation (SigmaPlot v. 12.0).

### 3.4. Identification of NG peptide precursors and neuropeptide-S/crustacean cardioactive peptide-type receptors in echinoderms

In addition to the NGFFFamide precursor in *S. purpuratus* (class Echinoidea), precursors of NG peptides have also been identified in other echinoderms. These include the precursor of NGIWYamide in the sea cucumber *Apostichopus japonicus* (class Holothuroidea) [22] and the precursor of NGFFYamide in the starfish *Asterias rubens* (class Asteroidea) [23]. Here, we sought to identify candidate NPS/CCAP-type receptors for these peptides. We also sought to identify precursors of NG peptides and NPS/CCAP-type receptors in species belonging to the two other extant echinoderm classes: class Ophiuroidea (the brittle star *Ophionotus victoriae*) and class Crinoidea (the feather star *Antedon mediterranea*). Transcriptome sequence data from *A. japonicus* [24], *A. rubens* [23], *O. victoriae* [25] and *A. mediterranea* [25] were subject to BLAST analysis using SequenceServer (http://www.sequenceserver.com), with the *S. purpuratus* NGFFFamide precursor and *S. purpuratus* NPS/CCAP-type receptor employed as protein query sequences.

## 4. Results

### 4.1. Mass spectrometric detection of NGFFFamide in extracts of the sea urchin *Strongylocentrotus purpuratus*

To test the hypothesis that NPS/CCAP-type receptors in deuterostomian invertebrates are activated by NG peptides, the *S. purpuratus* NPS/CCAP-type receptor was selected as a model system. Therefore, we first had to establish that its putative ligand, NGFFFamide, is present in *S. purpuratus*. Indirect evidence that NGFFFamide is derived from the NG peptide-type precursor in sea urchins has been provided by the discovery that synthetic NGFFFamide is a potent stimulator of muscle contraction in the sea urchin *Echinus esculentus* [26]. However, definitive proof that NGFFFamide exists in *S. purpuratus* required use of mass spectrometry. A peptide with the expected molecular mass for protonated NGFFFamide (630.3) was detected in extracts of *S. purpuratus* and its chromatographic properties were identical to synthetic NGFFFamide (electronic supplementary material, figure S1). These data confirmed that NGFFFamide is a naturally occurring peptide in *S. purpuratus*.

### 4.2. Cloning and sequencing of a cDNA encoding the *Strongylocentrotus purpuratus* neuropeptide-S/crustacean cardioactive peptide-type receptor

The occurrence of a gene in *S. purpuratus* encoding an NPS/CCAP-type receptor has been reported previously [8,27,28], and transcriptome sequencing [29] has confirmed the sequence of a predicted 444-residue protein. To enable pharmacological characterization of this receptor, a cDNA encoding the receptor was cloned and sequenced using total cDNA from 72 h old *S. purpuratus* embryos. Several polymorphisms at the nucleotide level were identified, but the sequence of the predicted 444-residue protein was identical to previous reports based on genomic/transcriptomic sequence data (electronic supplementary material, figure S2; GenBank accession number: KP171538).

### 4.3. NGFFFamide is a potent activator of the *Strongylocentrotus purpuratus* neuropeptide-S/crustacean cardioactive peptide-type receptor

**Figure 1.**
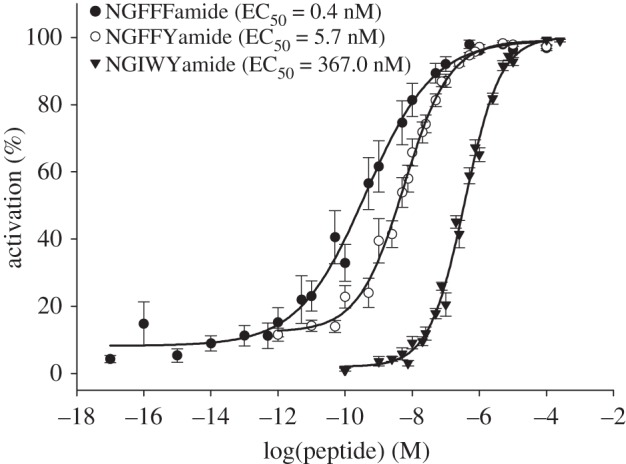
NG peptides are ligands for the *S. purpuratus* NPS/CCAP-type receptor. Dose–response curves for Ca^2+^ responses evoked by the NG peptides NGFFFamide (*S. purpuratus*), NGFFYamide (*A. rubens*) and NGIWYamide (*A. japonicus*) in CHO-K1 cells expressing the *S. purpuratus* NPS/CCAP-type receptor. Each point (±s.e.m.) represents mean values from at least two independent experiments performed in at least triplicate. Dose–response data are shown as relative (%) to the highest value (100% activation) after normalization to the maximum Ca^2+^ response. The log EC_50_ values (±s.e.m.) are NGFFFamide: −9.38 ± 0.09; NGFFYamide: −8.25 ± 0.04; NGIWYamide: −6.44 ± 0.04.

To determine whether NGFFFamide is the cognate ligand for the *S. purpuratus* NPS/CCAP-type receptor, the receptor was expressed in CHO-K1 cells stably overexpressing the human Gα_16_ protein and mitochondrially targeted apo-aequorin to assay Ca^2+^ levels. Control experiments on cells transfected with an empty vector revealed no effect of NGFFFamide on Ca^2+^ levels (data not shown). However, in cells transfected with the *S. purpuratus* NPS/CCAP-type receptor, NGFFFamide caused dose-dependent Ca^2+^ elevation, with an EC_50_ of 0.4 nM (figure 1). To characterize the structure–activity relationships of NGFFFamide as a ligand for the *S. purpuratus* NPS/CCAP-type receptor, two structurally related NG peptides were tested: NGFFYamide from *A. rubens* [23] and NGIWYamide from *A. japonicus* [22,30]. Both peptides activated the receptor dose-dependently, but with lower potency than NGFFFamide; the EC_50_ value for NGFFYamide was 5.7 nM, and the EC_50_ value for NGIWYamide was 367 nM (figure 1). Interestingly, the 10-fold difference in the potency of NGFFFamide and NGFFYamide can be attributed to the occurrence of a single hydroxyl group in the C-terminal tyrosine of NGFFYamide. The additional 10-fold reduction in potency seen with NGIWYamide can be attributed to the more substantive differences in the side-chain structures of the isoleucine–tryptophan dipeptide in NGIWYamide and the phenylalanine–phenylalanine dipeptide in NGFFYamide.

### 4.4. Gene encoding the NGFFFamide receptor is located adjacent to a gene encoding a vasopressin/oxytocin-type receptor in the genome of *Strongylocentrotus purpuratus*

Having demonstrated that NGFFFamide activates the *S. purpuratus* NPS/CCAP-type receptor, we observed that the gene encoding this receptor is located adjacent to a gene encoding a VP/OT-type receptor in the *S. purpuratus* genome (scaffold 114 [27]; electronic supplementary material, figure S3). Furthermore, analysis of the phylogenetic distribution of NPS/CCAP-type receptors and VP/OT-type receptors indicates that the gene duplication event that gave rise to these paralogous receptor types occurred in a common ancestor of the Bilateria [8]. However, in other bilaterian species for which genome sequence data are available, including other deuterostomian invertebrates (e.g. *B. floridae*), the genes encoding NPS/CCAP-type receptors, and VP/OT-type receptors do not occupy adjacent positions (our unpublished data, 2015). Therefore, the adjacent location of the genes encoding these two receptor types in the genome of *S. purpuratus* is remarkable because it presumably reflects conservation of the adjacent positioning of genes generated by a tandem gene duplication event that occurred approximately 600 million years ago in a common ancestor of the Bilateria.

### 4.5. Discovery of novel neuropeptide-S/crustacean cardioactive peptide-type receptors and candidate ligands derived from NG peptide-type precursors in deuterostomian invertebrates

Having demonstrated that NGFFFamide is a cognate ligand for the NPS/CCAP-type receptor of the sea urchin *S. purpuratus* (phylum Echinodermata), we then sought to identify genes/transcripts encoding NG peptide-type precursors and NPS/CCAP-type receptors in other deuterostomian invertebrates. This was of interest to facilitate reconstruction of the evolution of NG peptide signalling systems in the deuterostomian branch of the animal kingdom and to predict the molecular identity of the ligands of NPS/CCAP-type receptors in deuterostomian invertebrates.

Previous studies have reported the co-occurrence of genes encoding NG peptide precursors and genes encoding NPS/CCAP-type receptors in species belonging to other deuterostomian phyla/subphyla—the acorn worm *S. kowalevskii* (phylum Hemichordata) and the lancelet *B. floridae* (phylum Chordata; subphylum Cephalochordata) [8,28]. Interestingly, in the sea squirt *Ciona intestinalis* (phylum Chordata; subphylum Urochordata), an NPS/CCAP-type receptor gene is not present, and in accordance with this finding a gene encoding an NG peptide-type precursor has also not been found in this species [8,28]. Consistent with this finding, our analysis of genome sequence data from another urochordate species, the pelagic tunicate *Oikopleura dioica* [31] (http://www.genoscope.cns.fr/externe/GenomeBrowser/Oikopleura/), reveals an absence of genes encoding an NPS/CCAP-type receptor and an NG peptide-type precursor. Thus, it appears that loss of a gene encoding an NPS/CCAP-type receptor is associated with loss of an NG peptide-type precursor gene or vice versa, a finding that is consistent with our discovery that NGFFFamide is the ligand of the NPS/CCAP-type receptor in the sea urchin *S. purpuratus*.

Here, we have extended phylogenetic analysis of NPS/CCAP-type receptors and NG peptide-type precursors in the phylum Echinodermata beyond the sea urchin *S. purpuratus* (class Echinoidea) by analysing transcriptome sequence data obtained from representative species belonging to the four other extant classes of this phylum: the Holothuroidea (the sea cucumber *A. japonicus*), the Asteroidea (the starfish *A. rubens*), the Ophiuroidea (the brittle star *O. victoriae*) and the Crinoidea (the feather star *A. mediterranea*).

**Figure 2.**
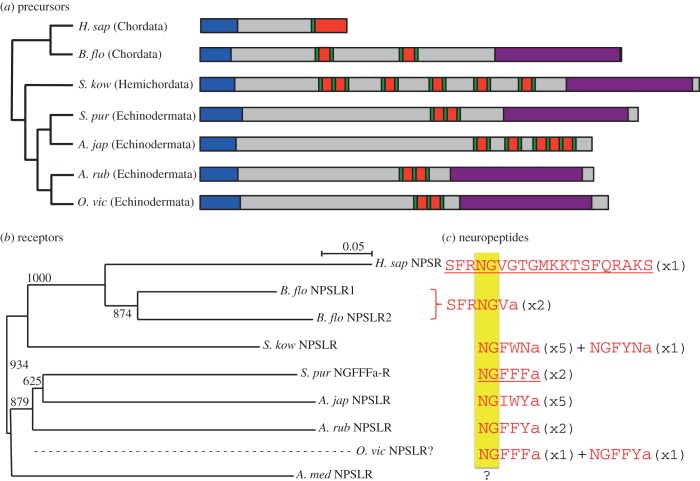
The NG peptide/NPS signalling system in deuterostomes. (*a*) Phylogenetic diagram comparing the structural organization of neuropeptide-S (NPS) and NG peptide precursors in deuterostomes. Signal peptides are shown in blue, neuropeptides are shown in red (bounded by dibasic cleavage sites in green), and neurophysin domains are shown in purple. (*b*) Neighbour-joining tree (including bootstrap values out of 1000) showing NPS-type receptors in deuterostomes, with the hypothetical phylogenetic position of an as yet unidentified NPS-type receptor in the brittle star *O. victoriae* represented by the dashed line. (*c*) Alignment of neuropeptide(s) derived from the NPS/NG peptide precursors shown in (*a*); these neuropeptides are proven (underlined) or candidate (not underlined) ligands for the corresponding receptors shown in (*b*). The conserved NG motif is highlighted in yellow and the numbers in parentheses represent the number of copies of the peptide in the precursor. *H. sap, Homo sapiens*; *B. flo, Branchiostoma floridae*; *S. kow, Saccoglossus kowalevskii*; *S. pur, Strongylocentrotus purpuratus*; *A. jap, Apostichopus japonicus*; *A. rub, Asterias rubens*; *O. vic, Ophionotus victoriae*; *A. med*, *Antedon mediterranea*.

The Holothuroidea are a sister class to the Echinoidea in a clade of echinoderms known as the Echinozoa [32,33]. In the sea cucumber *A. japonicus*, a transcript encoding an NG peptide-type precursor protein comprising five copies of the neuropeptide NGIWYamide has been identified, but, interestingly, unlike the sea urchin NGFFFamide precursor, it lacks a C-terminal neurophysin domain [22,23] (figure 2*a*). Here, our BLAST analysis of *A. japonicus* transcriptome sequence data using the *S. purpuratus* NGFFFamide receptor sequence as a query has identified a candidate NPS/CCAP-type receptor for NGIWYamide (figure 2*b*; electronic supplementary material, figure S4).

The Asteroidea (starfish) and Ophiuroidea (brittle stars) are sister classes in the asterozoan clade of the phylum Echinodermata [32,33]. Previously, we have reported discovery of an NG-peptide-type precursor in the starfish *A. rubens* that is very similar to the sea urchin NGFFFamide precursor—it comprises two copies of the peptide NGFFYamide and a C-terminal neurophysin domain [23]. Here, we have identified a transcript encoding an NPS/CCAP-type receptor in *A. rubens*, which is a candidate receptor for the neuropeptide NGFFYamide (figure 2*b*; electronic supplementary material, figure S5; GenBank accession number: KP171535). Hitherto, NG peptide-type precursors have not been identified in species belonging to the class Ophiuroidea, but here we have identified a transcript encoding an NG peptide-type precursor in the brittle star species *O. victoriae* (figure 2*a*; electronic supplementary material, figure S6; GenBank accession number: KP171536). The precursor is similar to the sea urchin NGFFFamide precursor and the starfish NGFFYamide precursor—it has a C-terminal neurophysin domain and two NG peptides. Interestingly, one of the NG peptides is NGFFFamide (as found in *S. purpuratus*), and the other NG peptide is NGFFYamide (as found in *A. rubens;* figure 2*c*). However, analysis of our *O. victoriae* transcriptome sequence data did not reveal a candidate NPS/CCAP-type receptor for these peptides.

Basal to the Echinozoa and the Asterozoa in the phylum Echinodermata is the class Crinoidea, which includes sea lilies and feather stars [32,33]. Analysis of transcriptome sequence data from the feather star *A. mediterranea* identified an NPS/CCAP-type receptor in this species that is an orthologue of the *S. purpuratus* NGFFFamide receptor (figure 2*b*; electronic supplementary material, figure S7; GenBank accession number: KP171537). However, we were unable to identify a candidate NG peptide-type ligand for this receptor in *A. mediterranea*. Thus, when the *A. mediterranea* transcriptome sequence data were subject to BLAST analysis using the NGFFFamide precursor as a query, the only transcript that exhibited significant similarity to the NGFFFamide precursor was a VP/OT-type precursor (data not shown). Recognizing that there may have been loss of the C-terminal neurophysin domain in the putative *A. mediterranea* NG peptide precursor, as previously seen in the holothurian *A. japonicus*, we also searched for transcripts encoding proteins comprising peptide sequences with an NG peptide motif, but no candidate NG peptide precursors were identified.

## 5. Discussion

We have demonstrated that an orthologue of vertebrate NPS-type receptors in the sea urchin *S. purpuratus* is activated by NGFFFamide, a neuropeptide that is derived from a neurophysin-containing precursor protein. This finding unites a bilaterian family of neuropeptides that includes NPS-type peptides in vertebrates, NG peptides in deuterostomian invertebrates and CCAP-type neuropeptides in protostomian invertebrates. Furthermore, it provides definitive support for a scenario of neuropeptide-receptor evolution that has been postulated based on phylogenetic reconstruction of bilaterian neuropeptide systems [8,28]. Thus, our experimental demonstration of the NG peptide–NPS/CCAP-type receptor signalling system provides the missing link that enables an understanding of the relationship of NPS with CCAP and their shared ancestry with VP/OT-type neuropeptides. The sea urchin NGFFFamide receptor is the first neuropeptide receptor to be characterized in an echinoderm, and our findings illustrate the importance of studies on echinoderms (and other deuterostomian invertebrates) in enabling reconstruction of the evolutionary history of neural signalling systems.

**Figure 3.**
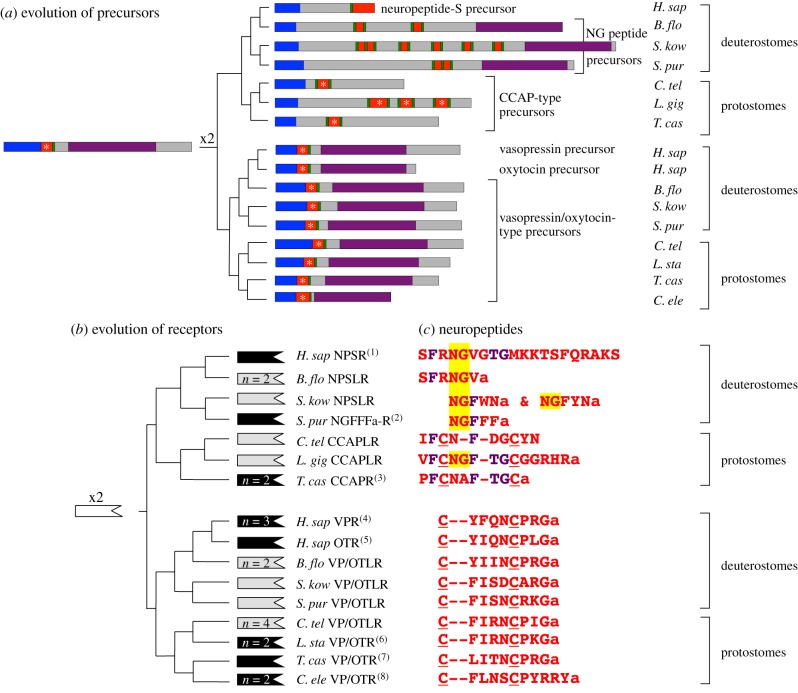
Evolution of NPS/NG peptide/CCAP-type and vasopressin/oxytocin-type signalling systems in the bilateria. (*a*) Schematic showing how duplication of a vasopressin (VP)/oxytocin (OT)-type neuropeptide precursor in a common ancestor of the bilateria gave rise to the highly conserved precursors of VP/OT-type neuropeptides and the divergent precursors of NPS, NG peptides and CCAP-type peptides in extant bilaterians. Signal peptides are shown in blue, neuropeptides are shown in red (with the presence of a disulfide bridge labelled with an asterisk), dibasic cleavage sites are shown in green and neurophysin domains are shown in purple. (*b*) Schematic showing how duplication of a receptor in a common ancestor of the bilateria (white-filled receptor symbol) gave rise to NPS/NG peptide/CCAP-type receptors and VP/OT-type receptors in deuterostomian and protostomian species. Receptors where the molecular identity of the cognate ligand has been determined are shown as a black-filled receptor symbols and receptors where the molecular identity of the cognate ligand remains to be proven are shown as grey-filled receptor symbols. Where multiple isoforms of a receptor type occur in a species, this is shown as *n* = *x*. (*c*) Alignment of NPS/NG peptide/CCAP-type neuropeptides and VP/OT-type neuropeptides that are derived from the precursors shown in (*a*), which are proven or candidate ligands for the receptors shown in (*b*). An NG motif, which is highlighted in yellow, is a conserved feature of NPS, NG peptides and some CCAP-type peptides. A pair of cysteine residues (underlined), which form a disulfide bridge, are a conserved feature of CCAP-type peptides and VP/OT-type peptides. A TG motif and two phenylalanine (F) residues, which are conserved in sub-sets of peptides, are shown in purple. *H. sap, Homo sapiens*; *B. flo, Branchiostoma floridae*; *S. kow, Saccoglossus kowalevskii*; *S. pur, Strongylocentrotus purpuratus*; *C. tel, Capitella teleta*; *L. gig, Lottia gigantea*; *T. cas, Tribolium castaneum*; *L. sta, Lymnaea stagnalis*; *C. ele, Caenorhabditis elegans*. References: 1. [3]; 2. this paper; 3. [34]; 4. [35–37]; 5. [38]; 6. [39]; 7. [40]; 8. [21,41].

In figure 3, we present a diagrammatic representation of the evolutionary steps that gave rise, on the one hand, to NPS in humans and other vertebrates, and on the other hand, to the structurally unrelated neuropeptide CCAP in insects (e.g. *Drosophila* and *Tribolium castaneum*) and other protostomian invertebrates. Our findings support a scenario wherein duplication of a gene encoding a VP/OT-type precursor occurred in a common ancestor of the Bilateria (see x2 in figure 3*a*).

One copy retained the presumed ancestral features of the precursor, which can be seen in the highly conserved structure of VP/OT-type precursors in extant Bilateria—an N-terminal signal peptide, followed by a single copy of a VP/OT-type peptide and a much larger neurophysin domain. Thus, as illustrated in figure 3*a*, VP/OT-type precursors that share this conserved structural organization have been identified in species from many bilaterian phyla, although loss of VP/OT-type precursors has occurred in some lineages (e.g. *Drosophila* and other dipterans [34,42]).

The other gene copy diverged from the ancestral form, but divergence took different courses in the protostomian and the deuterostomian branches of the animal kingdom. In the protostomian lineage, the occurrence of a disulfide bridge in the neuropeptide product(s) of the precursor was retained, and in this respect, CCAP-type peptides in extant protostomian species are similar to VP/OT-type peptides (see asterisks in figure 3*a*). However, CCAP-type precursors do not have a C-terminal neurophysin domain, and therefore this feature of VP/OT-type precursors was lost during the evolutionary history of CCAP-type precursor genes. In the deuterostomian lineage, we see the opposite scenario. Thus, here, the neuropeptides lost a disulfide bridge, but the neurophysin domain was retained, and these features can be seen, for example, in the precursor of the neuropeptide NGFFFamide in the sea urchin *S. purpuratus*. Interestingly, however, the neurophysin domain was subsequently lost in the vertebrate lineage that gave rise to the NPS-type precursors in extant tetrapod vertebrates. Furthermore, the absence of NPS-type precursors in teleosts and basal vertebrate classes (Chondrichthyes, Agnatha) [6] indicates that there has also been independent loss of NG peptide/NPS-type precursor genes in multiple vertebrate lineages. However, the loss of NG peptide/NPS-type precursor genes is not unique to basal vertebrates; it is also a feature of urochordates. Furthermore, in some invertebrate deuterostome lineages, such as the holothurian echinoderms (sea cucumbers), NG peptide precursors have secondarily lost the neurophysin domain (see *A. jap*. in figure 2*a*). Lastly, additional evidence of the occurrence of an ancient gene duplication that gave rise to VP/OT-type precursors and NPS/NG peptide/CCAP-type precursors can be found in the genomes of extant animal species. Thus, in the cephalochordate *B. floridae*, the gene encoding a VP/OT-type precursor in this species is located in tandem with the gene that encodes the precursor of the putative NPS-like NG peptide in this species—SFRNGVamide [8,23].

A contentious issue concerns the structural organization of the ancestral precursor protein that gave rise to VP/OT-type precursors and NPS/NG peptide/CCAP-type precursors. In previously reported models of the evolution of these two neuropeptide families, it was proposed that the ancestral precursor would have comprised two neuropeptides—one copy of a VP/OT-type peptide and one copy of an NPS/NG peptide/CCAP-type peptide, together with a C-terminal neurophysin domain [8,28]. In our model (figure 3*a*), the ancestral precursor comprises only one neuropeptide, and we speculate that this would have been similar to VP/OT-type peptides and CCAP-type peptides in extant animals in having two cysteine residues that form a disulfide bridge. A key basis for this supposition is that the existence of one VP/OT-type neuropeptide and one neurophysin domain is a highly conserved feature of VP/OT-type neuropeptide precursors in extant Bilateria, and it seems likely, therefore, that this reflects conservation of an ancestral precursor structure. Furthermore, a biochemical basis for this 1 : 1 relationship can be seen in the stoichiometry of the interaction of VP/OT-type peptides with neurophysin, where a homodimeric neurophysin complex binds two VP/OT-type peptide molecules [18,43]. In the highly divergent NPS/NG peptide/CCAP-type neuropeptide precursors, there has been loss of the neurophysin domain in multiple lineages, and where the neurophysin domain has been retained it is in association with two to six copies of an NG peptide. The significance of the retention of the neurophysin domain in some, but not all NG peptide-type precursors in deuterostomes is unclear, but it is a fascinating issue for further investigation. Retention of the neurophysin domain suggests that it is functionally important for the biosynthesis of NG peptides. However, because the number of NG peptides in association with neurophysin in NG peptide precursors is variable (2 : 1; 5 : 1; 6 : 1—figures 2*a* and 3*a*), it remains unclear whether or not NG peptides actually bind to their associated neurophysin. If they do, then what is the stoichiometry of the interaction? If they do not, then what is the role of the neurophysin domain in NG peptide precursors?

In order that duplication of a gene encoding an ancestral VP/OT-type precursor could give rise to two functional peptide-receptor signalling systems, there would have to have been a contemporaneous duplication of the gene encoding the receptor for the putative ancestral VP/OT-type peptide. This gene duplication is portrayed as x2 in the dendrogram shown in figure 3*b*. As reported above for NG peptide precursor genes, additional evidence of the occurrence of an ancient gene duplication that gave rise to VP/OT-type receptors and NPS/NG peptide/CCAP-type receptors can be found in the genomes of extant animal species. Thus, in the sea urchin *S. purpuratus*, the gene encoding a VP/OT-type precursor is located in tandem with the gene encoding the NGFFFamide receptor that we have characterized pharmacologically in this study (electronic supplementary material, figure S3).

In figure 3*c*, we show sequence alignments of proven or candidate peptide ligands for the receptors in figure 3*b*. By comparing the sequences of NPS, NG peptides and CCAP-type peptides with each other and with the sequences of VP/OT-type peptides, a number of shared characteristics can be seen, which may be reflective of the common evolutionary origins of these neuropeptides. Comparison of CCAP-type peptides with VP/OT-type peptides highlights a common feature of two cysteine residues, which in the mature bioactive peptides form a disulfide bridge, as highlighted above. Interestingly, comparison within the NPS/NG peptide/CCAP group reveals that the NG motif is not only a feature of NPS and NG peptides in deuterostomes, as it is also a feature of CCAP-type neuropeptides in molluscs. This is illustrated in figure 3*c* with a CCAP peptide from the limpet *Lottia gigantea,* but the NG motif is also a feature of CCAP-type peptides in other mollusc species (e.g. *Conus villepinii*, GI:325529921; *Aplysia californica*, GI:524893759). Thus, the NG motif may be a unifying characteristic of the bilaterian family of neuropeptides that includes NPS, NG peptides and CCAP-type peptides, but with subsequent loss of the glycine residue or substitution of the glycine with another residue (alanine) in some CCAP-type peptides. A feature that appears to unify NPS with CCAP-type peptides is a Thr–Gly (TG) motif, which is absent in NG peptides of deuterostomian invertebrates (figure 3*c*). Lastly, alignment of two phenylalanine residues in CCAP-type neuropeptides with phenylalanines in NPS and NG peptides reveals what appear to be differential patterns of conservation in chordate and non-chordate deuterostomes. Thus, the phenylalanine residue at position two in CCAP peptides aligns with a phenylalanine residue at position two in NPS and in the NPS-like NG peptide of *B. floridae*, whereas the phenylalanine residue at position five or six in CCAP peptides aligns with a phenylalanine residue at position three in ambulacrarian NG peptides (figure 3*c*).

Our discovery that the NPS/CCAP-type receptor in the sea urchin *S. purpuratus* is activated by the NG peptide NGFFFamide provides a basis for investigation of the physiological roles of NG peptides in deuterostomian invertebrates. This will provide new insights into the evolution of the physiological and behavioural functions of NPS/NG peptide/CCAP-type neuropeptides in the animal kingdom. Based upon what is currently known, a common theme seems to be a role in control of behaviours that are associated with a heightened state of arousal. Thus, NPS causes anxiolysis and arousal in mammals [3], NG peptides trigger dynamic behaviours linked to feeding or reproduction in echinoderms [23,44], and CCAP activates the ecdysis motor programme in arthropods that results in shedding of the exoskeleton [11]. As we learn more about the physiological/behavioural roles of both NPS/NG peptide/CCAP-type neuropeptides and VP/OT-type neuropeptides in a variety of phyla, it may be possible to reconstruct how the highly conserved VP/OT-type and the highly divergent NPS/NG peptide/CCAP-type neuropeptide signalling systems acquired distinct physiological roles following ancient gene duplication events in the common ancestor of the Bilateria.

## Supplementary Material

Supplementary Material

## Data accessibility

Novel nucleotide sequence data have been deposited in the GenBank database (accession numbers KP171535–KP171538). The datasets supporting this article have been uploaded as part of the electronic supplementary material. NG peptide precursor sequences, the NGFFFamide receptor sequence and other putative NG peptide receptor sequences have also been included in FASTA format in electronic supplementary material, figures S8 and S9.
