# Supplementary material for: Discovery of sea urchin NGFFFamide receptor unites a bilaterian neuropeptide family
Source: Open Biol. 2015 Apr 22;5(4):150030. doi: 10.1098/rsob.150030 (PMC4422128; doi:10.1098/rsob.150030)
Supplement: Supplementary Material [file rsob150030supp1.pdf]

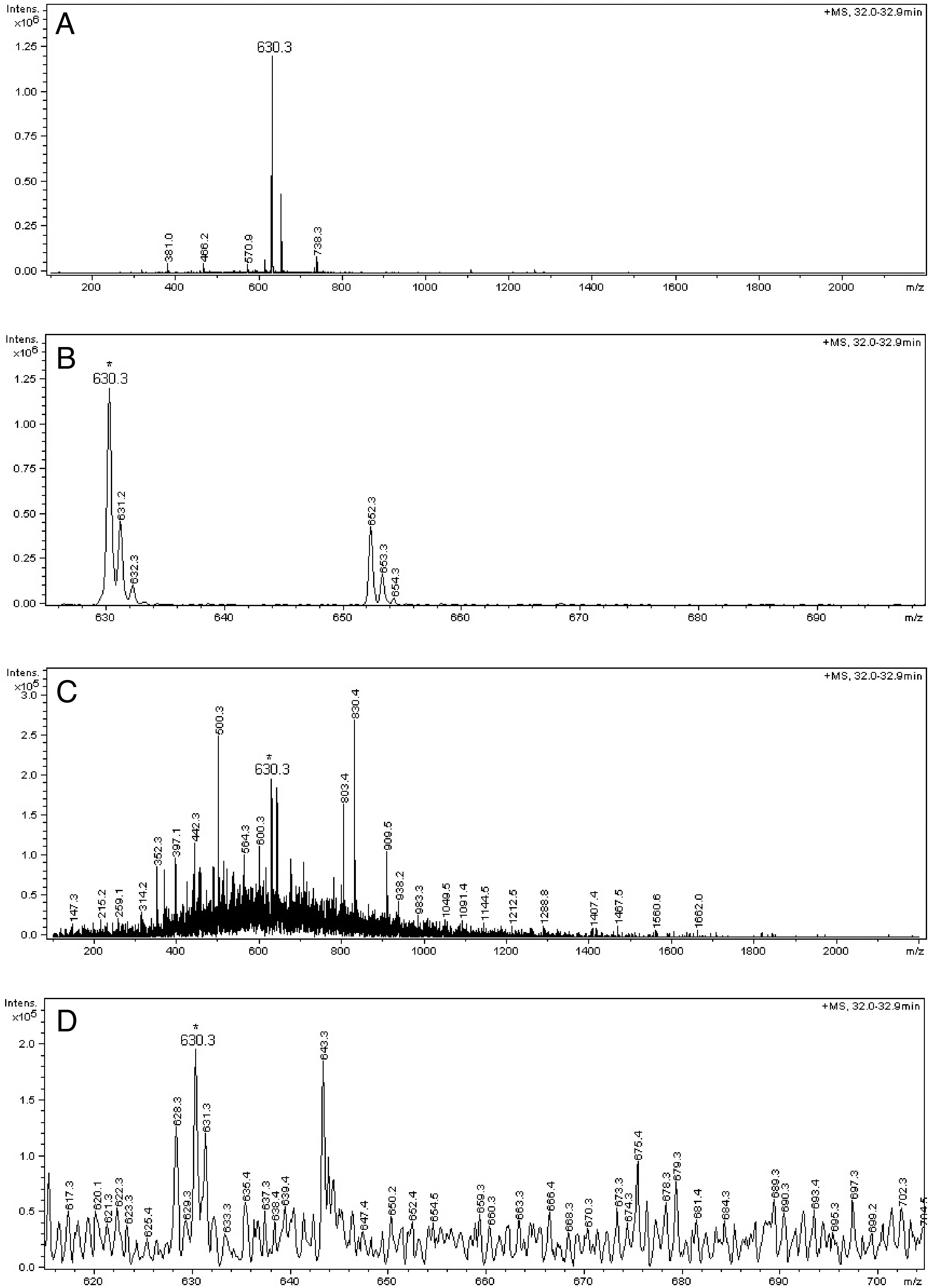
A
Intens.
x10^6
+MS, 32.0-32.9min
630.3
381.0
466.2
570.9
738.3
m/z
B
Intens.
x10^6
+MS, 32.0-32.9min
*
630.3
631.2
632.3
652.3
653.3
654.3
m/z
C
Intens.
x10^5
+MS, 32.0-32.9min
500.3
830.4
*
630.3
803.4
442.3
397.1
352.3
564.3
600.3
909.5
147.3
215.2
259.1
314.2
938.2
983.3
1049.5
1091.4
1144.5
1212.5
1288.8
1407.4
1467.5
1560.6
1662.0
m/z
D
Intens.
x10^5
+MS, 32.0-32.9min
*
630.3
643.3
628.3
631.3
675.4
679.3
617.3
620.1
621.3
622.3
623.3
625.4
629.3
633.3
635.4
637.3
638.4
639.4
647.4
650.2
652.4
654.5
659.3
660.3
663.3
666.4
668.3
670.3
673.3
674.3
678.3
681.4
684.3
689.3
690.3
693.4
695.3
697.3
699.2
702.3
704.5
m/z

Fig. S1. Detection of NGFFFamide in an extract of *Strongylocentrotus purpuratus* body tests using HPLC-MS. **A**. Full mass spectrum showing detection of synthetic NGFFFamide peptide (630.3) at 32.0 to 32.9 minutes, marked by an asterisk. **B**. Magnified view of the NGFFFamide peak in (A) showing peak characteristics. **C**. Full mass spectrum showing detection of NGFFFamide (630.3; at 32.0 to 32.9 minutes, marked by an asterisk) in an HPLC-separated extract of *S. purpuratus* body tests. **D**. Magnified view of the NGFFFamide peak in (C) showing peak characteristics.

1 cc
3 gacatagaaagtcatagttctactcgcattatggcgacacaagtgaactttgaccctggg
M A T Q V N F D P G 10
63 gtcacgacgacagagggcttcgattatacagagcctgggtcaaacaatggcaccagtaac
V T T T E G F D Y T E P G S N N G T S N 30
123 ggcattgtcgacaggtggtcgcttgacaa**ac**acatccagcttgccgtcctctgggttctc
G I V D R W S L D K H I Q L A V L W V L 50
183 ttcaccctcatcatagtagggaacggcatcgtcctcatcgcaatatggctcgtccgccac
F T L I I V G N G I V L I A I W L V R H 70
243 aagaaatccagactcaacttcttcatcacaaatctcgccgttgcg**ga**tatctgtgtgggt
K K S R L N F F I T N L A V A D I C V G 90
303 ttattcagcgtgggttttgatattcttgatcgccagacaccggaatttataggcggtgac
L F S V G F D I L D R Q T P E F I G G D 110
363 atagcctgcaaactatatcgatatgtcca**ag**cttatgttgtgttggcttcatcgtaccag
I A C K L Y R Y V Q A Y V V L A S S Y Q 130
423 cttgtagcattgagttttgatcgattctttgctatagtttacccgatggatttcacaggc
L V A L S F D R F F A I V Y P M D F T G 150
483 aat**gg**taagcggtcgacgatgctggcagcgggcggttggatattaccagcggtgctaggt
N G K R S T M L A A G G W I L P A V L G 170
543 attacttcacccgtcgtttttcaagtcgatcccctagcttcaccagacgggacacaaatg
I T S P V V F Q V D P L A S P D G T Q M 190
603 gtgatgtcatgctggcctgctgcactctacagcaatcgatcgtggatactcaa**gg**tttac
V M S C W P A A L Y S N R S W I L K V Y 210
663 gcgatgtacgtcacctcctcgttcttctacattcctctgatactcatcaccttttgttac
A M Y V T S S F F Y I P L I L I T F C Y 230
723 gtcacaatcatcgtaaccatctggacgagagcgaagaagatgggcggaccccaaaaggtc
V T I I V T I W T R A K K M G G P Q K V 250
783 aagaagtcgaaaaacgcaaacagagat**gt**cgcctacgaaggattgtcaaaagacagcaac
K K S K N A N R D V A Y E G L S K D S N 270
843 tccacaatgcctaaacatcgagccagttcacgaggcctaatcccaagggctaagattaag
S T M P K H R A S S R G L I P R A K I K 290
903 accatcaaaatgaccatctgtatcgtctgt**gc**ttacatatgttgcttcatgccgttctct
T I K M T I C I V C A Y I C C F M P F S 310
963 ctcttctacacgctggaggcattcggatgcatagacacctccagccaagcggtcctcttg
L F Y T L E A F G C I D T S S Q A V L L 330
1023 gccacacccgttctccaaaacctcccatccctcaacagcgccaccaaccctttcatttat
A T P V L Q N L P S L N S A T N P F I Y 350
1083 ggcattttcagcaccaatgtctgcaaagaattga**ga**cggatacccgccatcaactggata
G I F S T N V C K E L R R I P A I N W I 370
1143 gccgacaaggtgccgtgctgcagcgcttggaaaccgctccgattcggccgtcccacctac
A D K V P C C S A W K P L R F G R P T Y 390
1203 cagaccaacacacacacgacggaattcaacaacttctccgacggccatacgggctcaaga
Q T N T H T T E F N N F S D G H T G S R 410
1263 gggcgcaacatcgtcagcatgagcgggaaggtggtcgatggcccttcccgggacgatagt
G R N I V S M S G K V V D G P S R D D S 430
1323 cgttccagggacgatagccgaaattcgactactagtccgatgtagtacggtgtcgtccgc
R S R D D S R N S T T S P M * 444
1383 aatgtcatcaacgacggtttatcctacacgatgtcggtggatggtatttggaccattatc
1443 caattattgtagcatttttctttttacaacagggtagactttcgtttgtggaactgctat
1503 atctgatctgac

Fig. S2. *Strongylocentrotus purpuratus* NGFFFamide receptor. The cDNA nucleotide sequence (lowercase, 1514 bases) encoding the NGFFFamide receptor protein (uppercase, 444 amino acid residues) is shown. The positions of introns in the gene encoding this protein are shown by highlighting the pairs of bases (bold and underlined) in the sequence that are interrupted by an intron. The asterisk shows the position of the stop codon. This sequence has been deposited in the GenBank database under accession number KP171538.

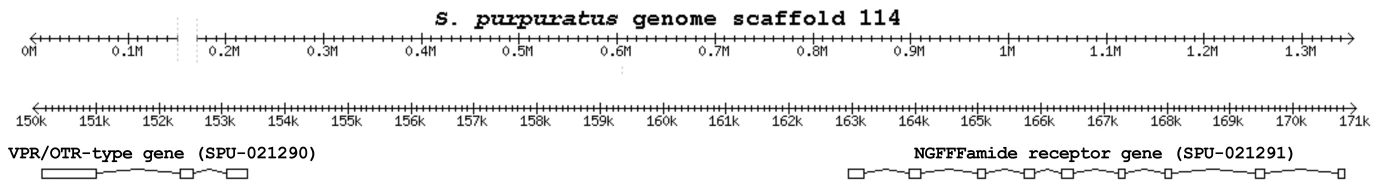

Fig. S3. Diagram showing that a vasopressin/oxytocin-type receptor gene (SPU_021290) is located adjacent to the NGFFFamide receptor gene (SPU_021291) on scaffold 114 of the *Strongylocentrotus purpuratus* genome. Exons are represented by white-filled rectangles and introns are represented by the lines. The diagram was generated as an output from the *Strongylocentrotus purpuratus* genome browser, which can be accessed at: http://www.spbase.org/cgi-bin/gb2/gbrowse/chado3.1/.

```
1                                                              ca
3    ctgattgatcgaaacaaaatggcggacgaaccagctgtactatcaacgctcggcaccgaa
                        M  A  D  E  P  A  V  L  S  T  L  G  T  E   14
63   ttacagaggacttcggaaaataccttcacgacaatcggaagccatgagcacgataacaga
      L  Q  R  T  S  E  N  T  F  T  T  I  G  S  H  E  H  D  N  R   34
123  ctggctgttgaatcggaactacaattagctatactgtggacgatgtacattcttatagtg
      L  A  V  E  S  E  L  Q  L  A  I  L  W  T  M  Y  I  L  I  V   54
183  gtcggtaacggtctggtcttacttgcgttgttcagtgtccggcacaagaaatcgagactg
      V  G  N  G  L  V  L  L  A  L  F  S  V  R  H  K  K  S  R  L   74
243  aacttcttcgtaaaacatttggcgatagcagacgtgtgtgtcggactgttgaacgtttta
      N  F  F  V  K  H  L  A  I  A  D  V  C  V  G  L  L  N  V  L   94
303  cccgagattatccatcgatacagaggtgcattttatgccggaatgttcctgtgcaaaatc
      P  E  I  I  H  R  Y  R  G  A  F  Y  A  G  M  F  L  C  K  I  114
363  aagtcctatggtcaggcgtttgtgatatatgcctcgatttatcaaatggtggcgctgagc
      K  S  Y  G  Q  A  F  V  I  Y  A  S  I  Y  Q  M  V  A  L  S  134
423  ttggatcgtttctttgcgattgtctttcctatggattttatggccagccgaaagagatca
      L  D  R  F  F  A  I  V  F  P  M  D  F  M  A  S  R  K  R  S  154
483  acgtttatggcggcaggggcttggattttaccggggatgttagctacaccttctctggcc
      T  F  M  A  A  G  A  W  I  L  P  G  M  L  A  T  P  S  L  A  174
543  atttttgtaactgcggagctccatggtcaaccacagtgtgcaccaatagctctcctggag
      I  F  V  T  A  E  L  H  G  Q  P  Q  C  A  P  I  A  L  L  E  194
603  gacaagttgaagtaccagttgtattcactctatatcgttagtattacattcctggtaccg
      D  K  L  K  Y  Q  L  Y  S  L  Y  I  V  S  I  T  F  L  V  P  214
663  ctgatgattctctgcgtgtgttacggaaccatgattagcgtgatttggagacgaggaaaa
      L  M  I  L  C  V  C  Y  G  T  M  I  S  V  I  W  R  R  G  K  234
723  gcaatggcgccacccgtgaaatctgacaagaacgcaaactctggcggagtgaaatatacg
      A  M  A  P  P  V  K  S  D  K  N  A  N  S  G  G  V  K  Y  T  254
783  ggtttacagaaaaaggccaaacaagacgagaataacttcaaacatcgatctagttctcgt
      G  L  Q  K  K  A  K  Q  D  E  N  N  F  K  H  R  S  S  S  R  274
843  ggtttgattccgagagccaagataaagaccgttaagatgactatttgtattgtaattgct
      G  L  I  P  R  A  K  I  K  T  V  K  M  T  I  C  I  V  I  A  294
903  tacattctttgctggctgcccactagtttgtattttaccttggaggcattcaaggtggtt
      Y  I  L  C  W  L  P  T  S  L  Y  F  T  L  E  A  F  K  V  V  314
963  aaacccagtgcagatcctcaacatgctatttactgggtcagtgtcatcatgcagaatcta
      K  P  S  A  D  P  Q  H  A  I  Y  W  V  S  V  I  M  Q  N  L  334
1023 gtttatctgaacagcgccacgaacccgttcatctacggcttttttcagttccaatatctgc
      V  Y  L  N  S  A  T  N  P  F  I  Y  G  F  F  S  S  N  I  C  354
1083 aaagaactaagacgttattacatcatcagacaacttttaaaatggatgccatgttgcaag
      K  E  L  R  R  Y  Y  I  I  R  Q  L  L  K  W  M  P  C  C  K  374
1143 gttacagaacctggttatggacggtctactgccggcacggttatgacagaatttcacagc
      V  T  E  P  G  Y  G  R  S  T  A  G  T  V  M  T  E  F  H  S  394
1203 cacacggccgcaatatcggacaatcatcgtttcaaccacaacgcatccagtaacgacgaa
      H  T  A  A  I  S  D  N  H  R  F  N  H  N  A  S  S  N  D  E  414
1263 aaatccgtccgagaaacaagtcatatctgacaggaacagagtatgtgtgacgtataataa
      K  S  V  R  E  T  S  H  I  *                                 423
1323 gagctgtaacattcatcgagggaggagaaaattggaccaattaaaagaaggcagcagtgt
1383 gacgtaagacgacatttgtgacgtttgttagtagagtatcttcacgtttgtcagacgacg
1443 aaaaacgaagtgcggtcctaaccagcacgacgagatcacaacgatatcgctcgctgtacg
1503 aagtcgagaaagagcagtttaattcttaagatccggtccactggcgaccattagtatgca
1563 ggagtaacgagaaactgtactatttggttgtgagatcgtaatcgaagtggacacgatatc
1623 acgtagagtgtgtgtagaagggcg
```

Fig. S4. *Apostichopus japonicus* candidate NGIWYamide receptor (isotig 14190; Du et al., 2012). The nucleotide sequence (lowercase, 1646 bases) encoding the receptor protein (uppercase, 423 amino acid residues) is shown. The asterisk shows the position of the stop codon.

```
1                                                               at
3    gggtaacgtgcaagctgtgacttcaagatccaaaaaccattcagcaacgtcttcaatcaa
63   aaaatcttcaatgtgggaaatagagatgtattctgttcgaaatcgagatgagccaagttt
123  ctttgaagtgtgtgccagcgtttgctaaacatttaccttggtgcgttcaaacaaagacat
183  tatcagtccgtcatctccttcgtctgaacaaccaatgaagggagcttattcccactccta
243  aaaaccatggcaacaattccagcctatgaccacttagttactgactcggttatggcggga
           M  A  T  I  P  A  Y  D  H  L  V  T  D  S  V  M  A  G   18
303  tattcattgaacgatacagcctctactgtgatggttccgactggattaccgtcaacgcta
      Y  S  L  N  D  T  A  S  T  V  M  V  P  T  G  L  P  S  T  L   38
363  gagggcgcacccaacgcaacgacgtccgtcacttattttagtgatggggagaatagattg
      E  G  A  P  N  A  T  T  S  V  T  Y  F  S  D  G  E  N  R  L   58
423  tcattctatggggggttccagctgattgtgctctgggttctattcgggcttactgtgatt
      S  F  Y  G  G  F  Q  L  I  V  L  W  V  L  F  G  L  T  V  I   78
483  ggtaactccactgtactccttgccgtctacgtcatcagacacaagaagtctagactcaac
      G  N  S  T  V  L  L  A  V  Y  V  I  R  H  K  K  S  R  L  N   98
543  tttttcgtcgcccatcttgccgcatcagacctttttggttggtatcgtcaacaatggctat
      F  F  V  A  H  L  A  A  S  D  L  L  V  G  I  V  N  N  G  Y  118
603  gaagtgctatacagatacttaggagagttttatggcgggatggtcttctgtaaaatcatt
      E  V  L  Y  R  Y  L  G  E  F  Y  G  G  M  V  F  C  K  I  I  138
663  cgcttctcacaggcatatgtaataaacgcatcgtcatttcaactggttgccctgagtctg
      R  F  S  Q  A  Y  V  I  N  A  S  S  F  Q  L  V  A  L  S  L  158
723  gaccgtttttttgctattgtattcccgatggatttttctggaagtggtaagagggcgaat
      D  R  F  F  A  I  V  F  P  M  D  F  S  G  S  G  K  R  A  N  178
783  ttgatggcggtgacggcgtggatcgccccgttgttcgcctctataccatctgcgattgtt
      L  M  A  V  T  A  W  I  A  P  L  F  A  S  I  P  S  A  I  V  198
843  ttcgaggcggcagtggatagttgggggaaaacacattgtataccaccacctcttgtaccc
      F  E  A  A  V  D  S  W  G  K  T  H  C  I  P  P  P  L  V  P  218
903  ggaagttggcagtacaaggtgtacacgttatatgtggtgtctggtttcttctacatcccc
      G  S  W  Q  Y  K  V  Y  T  L  Y  V  V  S  G  F  F  Y  I  P  238
963  ttgatcattatcagcacgtgttatatattcatggttatctccatctggagaagaagcaag
      L  I  I  I  S  T  C  Y  I  F  M  V  I  S  I  W  R  R  S  K  258
1023 tacatgatggggcaaaaaccagaaaaagtcaaaggcaaagccgccaaaaacgcatctaag
      Y  M  M  G  Q  K  P  E  K  V  K  G  K  A  A  K  N  A  S  K  278
1083 gagaaggaaccgatgaaacacagagccagttcaagaggtttgatacccaaggcaaagatc
      E  K  E  P  M  K  H  R  A  S  S  R  G  L  I  P  K  A  K  I  298
1143 aagacgctcaagatgactgtctcaatcatcgttgcttttatcgtgtgttggtgtcccttc
      K  T  L  K  M  T  V  S  I  I  V  A  F  I  V  C  W  C  P  F  318
1203 tcagtattctacacattagatgcctttggagtgatctttatcgatgaagcaaaccttaac
      S  V  F  Y  T  L  D  A  F  G  V  I  F  I  D  E  A  N  L  N  338
1263 actgcattccgtgcgtcagccttcatccagaacttacctttctcaacagtgctatcaat
      T  A  F  R  A  S  A  F  I  Q  N  L  P  F  L  N  S  A  I  N  358
1323 cctttcatctatggaatgtttagcactaatatctgtcaagaactaagacgattcagcgtg
      P  F  I  Y  G  M  F  S  T  N  I  C  Q  E  L  R  R  F  S  V  378
1383 atcaactggatggcgacgaagctgcgttgctgtaaatcatggcggccctccaggtacgga
      I  N  W  M  A  T  K  L  R  C  C  K  S  W  R  P  S  R  Y  G  398
1443 cgctctactacactgcgtacagacactaacctgacagacatgtctgagggcgctagtggc
      R  S  T  T  L  R  T  D  T  N  L  T  D  M  S  E  G  A  S  G  418
1503 acacaccgaggacacactcgaccgttgtacgtgtacacaccaggaaggacgagcaacagt
      T  H  R  G  H  T  R  P  L  Y  V  Y  T  P  G  R  T  S  N  S  438
1563 gatcactccagggatctgtccaatagtgctatgtaattattaactatagcgcagggagca
      D  H  S  R  D  L  S  N  S  A  M  *                          449
1623 atacctgtcatggcagtataacgccactgggcttatgtatgactagcgcatgggccgtga
1683 gaccaatacctcttaccaccatgttctgtcctggcaacctaacgccattgggcttatata
1743 gtaagtaaaacggcctcacaggcgaacgttatttggtgaacgttgcgacctaaggctgtt
1803 taacactattccaatgtttttatccgcgacgggcaaaaagacaggtgattacttttcaaa
1863 atcagtgatttcattggatataacaatttcattggataaactagcagtgacgtaagctgt
1923 ctttgtaagttttggtttgtccgtggtgacattttgaagctgcacacggtcgtctgcata
1983 tgaatgtaggtg
```

Fig. S5. *Asterias rubens* candidate NGFFYamide receptor (contig 1116923). The nucleotide sequence (lowercase, 1994 bases) encoding the receptor protein (uppercase, 449 amino acid residues) is shown. The asterisk shows the position of the stop codon. This sequence has been deposited in the GenBank database under accession number KP171535.

```
1    ggcgcaataacggcggccagaaatcctggtcatttggtggtcggcgacgacatggcagtt
                                                            M  A  V     3
61   ggaataagaaacgttattcttcaccttattttggtattatatgtagcaaggacaatattg
      G  I  R  N  V  I  L  H  L  I  L  V  L  Y  V  A  R  T  I  L    23
121  ggtgaaatagatacatacaaggttcgtcgatacacggatgggtcaccagtaaattcgaat
      G  E  I  D  T  Y  K  V  R  R  Y  T  D  G  S  P  V  N  S  N    43
181  cattggacacaaagtgataacattaacgaactacgaaaggagattttgcatcactaccc
      H  W  T  Q  S  D  N  I  N  E  L  R  K  E  I  F  A  S  L  P    63
241  gccgatctcccgggtattattcacaaatcaaagacacatgattcgaggagcggtcgacat
      A  D  L  P  G  I  I  H  K  S  K  T  H  D  S  R  S  G  R  H    83
301  tcagacgaattatcgaacttagaacaattaggcaatgtagcgggtaaagacggtgccaat
      S  D  E  L  S  N  L  E  Q  L  G  N  V  A  G  K  D  G  A  N   103
361  gatgcaaaagacaaattggcattatataactttatgtccaaacaagctacacggaataat
      D  A  K  D  K  L  A  L  Y  N  F  M  S  K  Q  A  T  R  N  N   123
421  ttgggagatgaattagataaaagaaacggattcttctttggaaaaagaaacgggttcttt
      L  G  D  E  L  D  K  R  N  G  F  F  F  G  K  R  N  G  F  F   143
481  tatggcaagagggatgttgaagccgttaacaatgaagactcgtgcgtgagatgtggccca
      Y  G  K  R  D  V  E  A  V  N  N  E  D  S  C  V  R  C  G  P   163
541  gagaaccgtggtcagtgtgttatgtttggaacctgttgtagtcctcagttcggatgttat
      E  N  R  G  Q  C  V  M  F  G  T  C  C  S  P  Q  F  G  C  Y   183
601  ttaatgactaaggaatcagaggcatgcatgtcaaaccacatcggaacagggtgtcgtaat
      L  M  T  K  E  S  E  A  C  M  S  N  H  I  G  T  G  C  R  N   203
661  ctggatatggcgccgcagtgcggtagcacaggagtgtgtgttgcgaaaggtgtttgctgt
      L  D  M  A  P  Q  C  G  S  T  G  V  C  V  A  K  G  V  C  C   223
721  tcaccgcaagatggcgcttgtcacatcgatgtcacatcgtgcttatcagataatacaact
      S  P  Q  D  G  A  C  H  I  D  V  T  S  C  L  S  D  N  T  T   243
781  atcgacaaatctttgtagctagttttattcactatcagctttcaggaaataattccata
      I  D  K  S  L  *                                             248
841  tatttattttattactaaatttgaattgatgcaaaagtttctgatgtaatggaatgagtt
901  tcatgttcataattcataataagtgagattgcttgcgaaatagtaatccgttcataaacc
961  atccatcattcatgaagaatatgttagtgctatcaaactggcattgtgatgtaatagagt
1021 aaaggattattgcttacatggtcatggtataaaccgataaccagcattattgcatatgtg
1081 tatgtaaaagataaatactctgtgcttttaaacctgttggcagctttgacggaaaacgat
1141 acaatttgcacgatataatgttacaattatacaattagcacccctacatcacattggctt
1201 gacgcgtcaaagtagcgagtttgaaatgaccacgatttacataatttgagattcttgatt
1261 cttaaaatctgagttcttggattcagagcgttacgtttatacacaacaatgtcgttagct
1321 gcacctttgacaacattgcattttcattcaagtgtttgtagacacaatataagttccatc
1381 gtatcattaaatcactaagtgttggatgataaagaaatattgaatttcacttcacttcaa
1441 ttatgtaaggtatcataagactggaagcaagaatatagttagtgggaagtaagataattg
1501 atattgtccacaatccataaatgaatttaaatagtatgtatggacaaatgatgatttcta
1561 atatcgtttaatactactactactactactactacta
```

Fig. S6. *Ophionotus victoriae* NGFFFamide/NGFFYamide precursor (contig 2050454). The nucleotide sequence (lowercase, 1597 bases) encoding the precursor protein (uppercase, 248 amino acid residues) is shown. The predicted signal peptide is shown in blue, in tandem copies of the sequences NGFFF and NGFFY are shown in red, with C-terminal glycine (G) residues that are putative substrates for amidation shown in orange. Putative dibasic cleavage sites are shown in green. The C-terminal region of the precursor comprises a neurophysin domain (purple), with fourteen cysteine residues (underlined) that are a characteristic and conserved feature of neurophysins. The asterisk shows the position of the stop codon. This sequence has been deposited in the GenBank database under accession number KP171536.

```
1                                                                ac
3     agcgagaaagtaagtccgatagattcacacattatcattgtccacaaccatccgttaggc
63    ttacctaatgaaaacctctcttgttagattgttttagacttgagacgaagtaaacgattt
123   actgcatagatttatcaagacgtatcggtagtaaacgctgtgttcgtgttctctcgccgt
183   ttctaaaataacatcggaacgctttattattttgattaagtagtgggaactttctcattt
243   agaagatgtttagattatatctttgaattgatatgccctttcacatataagtaccactgt
303   gcacagtgtttatagacgcattgcattatggattataatatggacaattatacagacttt
                                       M  D  Y  N  M  D  N  Y  T  D  F   11
363   tggaccgaaatgactcccaccacaaaagaggtatccaggattgtgctttatcgggattta
       W  T  E  M  T  P  T  T  K  E  V  S  R  I  V  L  Y  R  D  L   31
423   caattgatagtcttgtggtccctcttcatttcgattttgattggaaatggagcagtactt
       Q  L  I  V  L  W  S  L  F  I  S  I  L  I  G  N  G  A  V  L   51
483   tttgctttatacactgttcgacataaaaagtcaagaatgaatttctttgttatgaactta
       F  A  L  Y  T  V  R  H  K  K  S  R  M  N  F  F  V  M  N  L   71
543   gcaatttccgatataattgttggatttttgaggttttggtgcaacttattcatagagga
       A  I  S  D  I  I  V  G  F  F  E  V  L  V  Q  L  I  H  R  G   91
603   tacactgggacatgggaaactggtaacatcgcctgtaaacttgttaaatatattcaggcg
       Y  T  G  T  W  E  T  G  N  I  A  C  K  L  V  K  Y  I  Q  A  111
663   gcttcattgacagcatcatcctgccagttggtcgctctaagtgtggatagatatctggct
       A  S  L  T  A  S  S  C  Q  L  V  A  L  S  V  D  R  Y  L  A  131
723   attatatatcccatgaacttttctggaagaagtcgaagagcacatatgatggctgcggct
       I  I  Y  P  M  N  F  S  G  R  S  R  R  A  H  M  M  A  A  A  151
783   gcgtggatcacgccgtttataacatctattacgtcactgcacattttgaaatccgagaa
       A  W  I  T  P  F  I  T  S  I  T  S  L  H  I  F  E  I  R  E  171
843   catggtgaagggacacagtgttggatggtactggaaaatgagaacttgaaattaaaatta
       H  G  E  G  T  Q  C  W  M  V  L  E  N  E  N  L  K  L  K  L  191
903   tacacaatatatgtgatgtgtattttgttctttattccgctggttgtcatcttattctgc
       Y  T  I  Y  V  M  C  I  L  F  F  I  P  L  V  V  I  L  F  C  211
963   tactgctctataatcattactatctggaaaaagagtaaaatgatgggtccaaatattaaa
       Y  C  S  I  I  I  T  I  W  K  K  S  K  M  M  G  P  N  I  K  231
1023  ccagcaagtaacaaaaagggtaataattatgacaatttaaaagctgatgagggcagtcgt
       P  A  S  N  K  K  G  N  N  Y  D  N  L  K  A  D  E  G  S  R  251
1083  tcacatcgtgcaagttcaagaggacttattccaaaggcaaaagttaaaacgatttacatt
       S  H  R  A  S  S  R  G  L  I  P  K  A  K  V  K  T  I  Y  I  271
1143  actttaagcattgtaattgcattcgttctgtgttggagtccgttcatctttacatatttg
       T  L  S  I  V  I  A  F  V  L  C  W  S  P  F  I  F  T  Y  L  291
1203  ttagccgcattccaagtgattcagcctagtgccaaactaatggcagtcatcgctaattta
       L  A  A  F  Q  V  I  Q  P  S  A  K  L  M  A  V  I  A  N  L  311
1263  ccagccattaacagcgccgtcaatccgctcatctatggaatattcagtaccaatctatgc
       P  A  I  N  S  A  V  N  P  L  I  Y  G  I  F  S  T  N  L  C  331
1323  cgggagctgaaacgaattcctgttattaattggtttgcgggagtgttgccttgttgtact
       R  E  L  K  R  I  P  V  I  N  W  F  A  G  V  L  P  C  C  T  351
1383  gctcggaaaaaggcagaacctggttttactcgtgcaatgtacactaggacagaaaacaca
       A  R  K  K  A  E  P  G  F  T  R  A  M  Y  T  R  T  E  N  T  371
1443  aatatggactatagcgttactacatgcgaacaggactctgaacgcattaaacttggccaa
       N  M  D  Y  S  V  T  T  C  E  Q  D  S  E  R  I  K  L  G  Q  391
1503  acaagtaaataagtcttagcagattgctaacatttatcctttcaattttaagccaccgat
       T  S  K  *                                                   394
1563  tttagaatgttaataattatcgcaatgtttcagcagtgtatttctcttaagaaaactatt
1623  actttcgacgacgactaatctagatagaattataatataacaaaatatatttaaaacttg
1683  tgtacacttttgttttgtttctttttatttattttgcttctgctattgcatttttata
1743  ctgt
```

Figure S7. *Antedon mediterranea* candidate NG peptide receptor (contig 1796804). The nucleotide sequence (lowercase, 1746 bases) encoding the receptor protein (uppercase, 394 amino acid residues) is shown. The asterisk shows the position of the stop codon. This sequence has been deposited in the GenBank database under accession number KP171537.

```
>Branchiostoma floridae SFRNGVamide precursor
MMQTPIFLCSVVLVGAVCGQLSETNNFPQNGNRRLSPERSATVLRQFLHLEGAVGSPVSP
SDGRALETGDKRSFRNGVGKRRDSEEERLPQNRGATELKAEATIFSQNGDPHDEGAKAAS
EKRSFRNGVGKRTHFRIVADASLGGLDEPEALRQTGGDANSPSLSRDLWAEVQGKDDEQC
PACGSDGSGVCVLKGVCCRLDSGCVLRKDVCSSLPDRALCASLQYSATCRTDGKCVAPGV
CCRAADHSCFLDPECD
>Saccoglossus kowalevskii NGFWNamide/NGFYNamide precursor
MLRKINAVVFFLVAICTLSRATFGEDGMTEKQVLKLHKYWPKEDISELGSSSTSGDSGEN
EAVKMGFWAVDGKRNGFWNGKRNGFWNGKRNFDDLRIFGTNKKHSSINNQKRNGFWNGKR
NFDDFEIPEKKEQPHWRDEKKNGFWNGKRNFDNFKTDDLQYPSIEDKRNGFWNGKRNFEM
SMNKKASASTESEKRNGFYNGKRSVDNTMNISSYHYKESAKKCTTCGPGGKGQCVMYGVC
CSLEIGCSMLTKETEECTTSPLVGECGRSDVQCGNGGRCVANGVCCTKETQSCKIDQECN
VRW
>Strongylocentrotus purpuratus NGFFFamide precursor
MGYERRILRTLLSILIVLASFVTVYGERDSNFMQQKQFRNIVPSPLIQKWRENRMGPAEE
KTSNEQWRDELLSNLRNVLRKHNASPSSRSRDRTDITAYGLQEPMQQLPADVTADQLFIL
EGAVNSPRENYEEETPIDEDKRNGFFFGKRNGFFFGKRSDSDASSTKMDDDRLPKYESSG
SFDKCRPCGPGRQGRCVMVGTCCSPLFGCYLFTPEAAACMTEDVSPCQLNAPSCGLAGKC
VADGICCSAAEGACHLDPTCTSMSLN
>Apostichopus japonicus NGIWYamide precursor
MAVEAKIVSCLVCIWLTSTVYSQSNTGRTHDYGELSKAVDTFLDILMDEENFDDVNNIES
WETVLKEDINPKLRILAHVMRSLSSRPDTSSLREQVFPSDYISRYLQEILTDEQPFWDES
SPKLPSLQTPELDQIKASADERNNFWSDNPSHRPPEGIPFSAGEESKRNGIWYGKRKSSL
DGEAVKRNGIWYGKRSSPPVDDKRNGIWYGKRNGIWYGKRNGIWYGKRDDSLYSEEMM
>Asterias rubens NGFFYamide precursor
MTMGSRSLLVTIVITVVIPSIWAGAIAGAQTQKIRRESRESGKYWPNSVGISDQQLRQLL
AHSLADSYSTSGASHIRGGDGDAGYIYDSRDQVDDTGTNEEEGERVIGSEVTSRDSNPGT
SKRNGFFYGKRNGFFYGKRSASTPGNANEVTQCIPCGPQNNGQCVMFGTCCSYELGGCFF
LTEEALPCVTSKSSSLCELSGLPCGDEGYGRCVADSVCCLPQEGSCHINAECGGKMTFQ
>Ophionotus victoriae NGFFFamide/NGFFYamide precursor
MAVGIRNVILHLILVLYVARTILGEIDTYKVRRYTDGSPVNSNHWTQSDNINELRKEIFA
SLPADLPGIIHKSKTHDSRSGRHSDELSNLEQLGNVAGKDGANDAKDKLALYNFMSKQAT
RNNLGDELDKRNGFFFGKRNGFFYGKRDVEAVNNEDSCVRCGPENRGQCVMFGTCCSPQF
GCYLMTKESEACMSNHIGTGCRNLDMAPQCGSTGVCVAKGVCCSPQDGACHIDVTSCLSD
NTTIDKSL
```

Fig. S8 NG peptide precursors in FASTA format

>Branchiostoma floridae NPS/CCAP-type receptor 1
TEQLVTLWVLFAFIVVGNSLVLLVMWMERHKKTRMNFFITNLAIADLGAGLFNVLPDLVH
RFTVEWIAGDFMCKLIKYIQGGVLYGSTYVLVALSVDRYDAIIHPMRFAHDRKSKAMICV
AWGLAALFSIPSPVIFAQTKLDNGEWQCWATWPEDWYWIPYMTIVTTLVFFIPLVIISTC
YIFIVVKIWRRSKELVQERKFIGGKRLSLSQGIIPKAKIKTIKLSLAIITAFIACWSPYF
VFDMITNFSDLPESETKKRASLIIQNLPALNSAINPIIYGFFSTKNCGKFR
>Branchiostoma floridae NPS/CCAP-type receptor 2
NYLVTLQTEQLVTLWVLFVFIVVGNSLVLLIMWLERNKTSRMNFFILNLAVADLSAGLFN
VLPDIVHRFVVEWIAGNTLCKLVKYTQAVLLYASTYVLVAMSIDRYDAIVHPLQFVREHK
SKVMISVAWGLALLFSVPSPVIFAVRRQPNGEWQCWAEWPEDWYWVPYMTVVAAFVFFIP
LGIISICYIAIIVKIWKRGKGMAYEDHIPRSRASSGKNFYRIFHLIHINNGFTSRAKART
IKLSVAIILAFICCWSPYFLFDILDNYDALPDTQAKKEASLIIQNLPALNSAINPIIYGF
FSTKLYRKLR
>Saccoglossus kowalevskii NPS/CCAP-type receptor
MTSKLNEMKYAAVMGAFINTTVYNNTVNNSTYDIPEKHQSVLSMYQTEQLILLWFLFAFV
VIGNAIVLVSVCLVRHKKSRMNFFIMNLAIADLSVGLLNILPDIIHRYTREFYGGEIVCK
LVKYVQAIVVYGSTYQLVALSIDRYDAIVHPMNFSGNKRSMIMVISMWVVAFILAVPSPV
FFEETVLENGEVQCWIELPQTWVWKPYSVILAFLLFFIPLVIVTFCYSVIIYTIWRKSKM
MVPAKRRIFNEKNGDSRGLIPKAKIKTIKMTLCIVLSFIVCWSPFTLWFILEIYGHIPKN
DLTMTIHIIVQNLPSLNSATNPAIYGLFSTNICKELR
>Strongylocentrotus purpuratus NGFFFamide receptor
MATQVNFDPGVTTTEGFDYTEPGSNNGTSNGIVDRWSLDKHIQLAVLWVLFTLIIVGNGI
VLIAIWLVRHKKSRLNFFITNLAVADICVGLFSVGFDILDRQTPEFIGGDIACKLYRYVQ
AYVVLASSYQLVALSFDRFFAIVYPMDFTGNGKRSTMLAAGGWILPAVLGITSPVVFQVD
PLASPDGTQMVMSCWPAALYSNRSWILKVYAMYVTSSFFYIPLILITFCYVTIIVTIWTR
AKKMGGPQKVKKSKNANRDVAYEGLSKDSNSTMPKHRASSRGLIPRAKIKTIKMTICIVC
AYICCFMPFSLFYTLEAFGCIDTSSQAVLLATPVLQNLPSLNSATNPFIYGIFSTNVCKE
LRRIPAINWIADKVPCCSAWKPLRFGRPTYQTNTHTTEFNNFSDGHTGSRGRNIVSMSGK
VVDGPSRDDSRSRDDSRNSTTSPM
>Apostichopus japonicus NPS/CCAP-type receptor
MADEPAVLSTLGTELQRTSENTFTTIGSHEHDNRLAVESELQLAILWTMYILIVVGNGLV
LLALFSVRHKKSRLNFFVKHLAIADVCVGLLNVLPEIIHRYRGAFYAGMFLCKIKSYGQA
FVIYASIYQMVALSLDRFFAIVFPMDFMASRKRSTFMAAGAWILPGMLATPSLAIFVTAE
LHGQPQCAPIALLEDKLKYQLYSLYIVSITFLVPLMILCVCYGTMISVIWRRGKAMAPPV
KSDKNANSGGVKYTGLQKKAKQDENNFKHRSSSRGLIPRAKIKTVKMTICIVIAYILCWL
PTSLYFTLEAFKVVKPSADPQHAIYWVSVIMQNLVYLNSATNPFIYGFFSSNICKELRRY
YIIRQLLKWMPCCKVTEPGYGRSTAGTVMTEFHSHTAAISDNHRFNHNASSNDEKSVRET
SHI
>Asterias rubens NPS/CCAP-type receptor
MATIPAYDHLVTDSVMAGYSLNDTASTVMVPTGLPSTLEGAPNATTSVTYFSDGENRLSF
YGGFQLIVLWVLFGLTVIGNSTVLLAVYVIRHKKSRLNFFVAHLAASDLLVGIVNNGYEV
LYRYLGEFYGGMVFCKIIRFSQAYVINASSFQLVALSLDRFFAIVFPMDFSGSGKRANLM
AVTAWIAPLFASIPSAIVFEAAVDSWGKTHCIPPPLVPGSWQYKVYTLYVVSGFFYIPLI
IISTCYIFMVISIWRRSKYMMGQKPEKVKGKAAKNASKEKEPMKHRASSRGLIPKAKIKT
LKMTVSIIVAFIVCWCPFSVFYTLDAFGVIFIDEANLNTAFRASAFIQNLPFLNSAINPF
IYGMFSTNICQELRRFSVINWMATKLRCCKSWRPSRYGRSTTLRTDTNLTDMSEGASGTH
RGHTRPLYVYTPGRTSNSDHSRDLSNSAM
>Antedon mediterranea NPS/CCAP-type receptor
MDYNMDNYTDFWTEMTPTTKEVSRIVLYRDLQLIVLWSLFISILIGNGAVLFALYTVRHK
KSRMNFFVMNLAISDIIVGFFEVLVQLIHRGYTGTWETGNIACKLVKYIQAASLTASSCQ
LVALSVDRYLAIIYPMNFSGRSRRAHMMAAAAWITPFITSITSLHIFEIREHGEGTQCWM
VLENENLKLKLYTIYVMCILFFIPLVVILFCYCSIIITIWKKSKMMGPNIKPASNKKGNN
YDNLKADEGSRSHRASSRGLIPKAKVKTIYITLSIVIAFVLCWSPFIFTYLLAAFQVIQP
SAKLMAVIANLPAINSAVNPLIYGIFSTNLCRELKRIPVINWFAGVLPCCTARKKAEPGF

TRAMYTRTENTNMDYSVTTCEQDSERIKLGQTSK

Fig. S9 NPS/CCAP-type receptors in deuterostomian invertebrates
